# Ecological Restoration and Carbon Sequestration Regulation of Mining Areas—A Case Study of Huangshi City

**DOI:** 10.3390/ijerph19074175

**Published:** 2022-03-31

**Authors:** Qipeng Liao, Xinran Liu, Mingzhu Xiao

**Affiliations:** 1 School of Arts and Communication, China University of Geosciences, Wuhan 430074, China; liuxinran@cug.edu.cn (X.L.); xiao_mingzhu@cug.edu.cn (M.X.); 2 Faculty of Fine Art, University of Barcelona, 08028 Barcelona, Spain

**Keywords:** net primary productivity (NPP), CASA model, double carbon goals, mining area, Huangshi City

## Abstract

As an important carbon sink indicator, the vegetation net primary productivity (NPP) is key and helpful for understanding regional carbon sequestration and storage of mining areas. Systematic analysis of NPP of the ecological reconstruction process in mining areas can effectively contribute to local governments and related departments for making ecological decisions under the “double carbon goals” (“peak of carbon release” and “carbon neutrality”) and help to promote regional sustainable development. In this study, we used the CASA model to systematically assess the temporal and spatial evolution characteristics of NPP of Huangshi City from 1990 to 2018. Meanwhile, various scenarios were set up to study the effects of climate factors, landscape pattern evolution, and ecological restoration on regional carbon storage. Our results documented that (1) NPP of the study area an increasing trend from 1990–2018 shows and exhibits significant spatial heterogeneity; (2) the significant increase of NPP was mainly in the restored mining areas, indicating that the ecological restoration of mining areas can effectively improve the regional carbon sequestration capacity; (3) from 1990 to 2018, climate change released 0.136 TgC, while landscape pattern change contributed to carbon storage with 0.266 TgC; and (4) the restoration and reconstruction of vegetation in the mining areas is an important way to achieve carbon neutrality of Huangshi City in the future, and the changes of NPP varied among different ecological restoration modes.

## 1. Introduction

Environmental issues about climate change are extremely important for the human society development. Hence, with the reduction of carbon emissions and the improvement of carbon sink, it has become a consensus to slow down the rate of global warming. The 14th Five-Year Plan for National Economic and Social Development of the People’s Republic of China and the Outline of Vision 2035 mentioned that China strives to peak CO_2_ emissions by 2030 and strives to achieve carbon neutrality by 2060, which is the “double carbon goals” [1]. Increasing the carbon storage of terrestrial ecosystems is an important way to achieve the “double carbon goals” and is also important for the current study of global climate change [2]. Net primary productivity (NPP) refers to the ability of plants to sequester carbon per unit area and per unit time, which is expressed as the portion of organic carbon fixed by photosynthesis minus that consumed by respiration [3,4]. NPP not only reflects regional carbon sequestration but also could be used to identify ecosystem carbon sources/sinks, playing an important role in global change and carbon balance [5,6]. Previous NPP-related studies have long received extensive attention. Regional NPP estimation and spatio-temporal evolution studies can effectively indicate the changes in regional carbon sequestration and its response to human activities. It is helpful for ecosystem quality monitoring and ecological benefit evaluation. [7,8].

The mining area is a unique artificial, semi-artificial special terrestrial ecosystem with the mining operation area as its core [9]. It usually goes through three stages of “natural vegetation–mining–restoration”, and from a holistic perspective, the study of carbon dynamic system emphasizes the dynamic of carbon sink in different stages. However, the carbon sequestration of mining sites varies among human activities. Therefore, the carbon sink potential of mine sites has been improved by taking measures to restore and reconstruct mine ecosystems favorably, increasing the carbon storage of mining areas [10]. Until now, many scholars have studied the carbon sink of mining areas under different stages. Mark et al. analyzed mining areas in seven states in the United States and showed that mine reclamation would greatly enhance the regional carbon sink [11]; Raj et al. documented the carbon storage of mining areas after reclamation from the perspective of soil carbon sequestration [12]; Jitendra et al. investigated the carbon sequestration capacity of vegetation on the mine areas after reclamation in specific climatic zones [13]. Zhang et al. studied the effects of land use change and ecological reconstruction on the changes of carbon sink in mining areas [10]. Xu et al. reported that climate change could promote the NPP in the mining areas, while frequent mining activities would decrease NPP [14]. Wu et al. demonstrated that the carbon stocks in mining areas were mainly affected by mining activities, and excessive mining activities decreased carbon stocks, but land reclamation increased the productivity of the land and increased carbon stocks [15]. Some studies focused on carbon stock estimation, spatial distribution variability, and the impact of mine restoration on carbon sink, but there is a lack of exploration on the spatial and temporal evolution of carbon sequestration in the multi-stage of mining. Therefore, a systematic analysis of the changes of NPP during the ecological reconstruction of mining areas can provide scientific supports for governmental ecological protection decisions, regional ecological management, and the realization of the “double carbon goals” [16,17].

Ecosystem restoration and rehabilitation of mining areas can turn difficult-to-use brownfields into green lands with high ecological benefits, and at the same time, ecological restoration shifted from a source releasing carbon to a sink sequestering carbon. Although mining areas cover a small area at the urban scale or regional scale, the change from a carbon source to a carbon sink is an important way to achieve regional carbon stock enhancement. However, most of the studies on the carbon sink in mining areas have focused on the field scale. The analysis of the spatial and temporal evolution of the carbon sink at the regional scale and the research on the ecological restoration effect of the mine area are still unclear. The effects of vegetation restoration on carbon storage in the whole study area have not been effectively revealed, and the multi-factor coupling analysis of carbon sequestration in mining areas is insufficient. Therefore, estimating the effects of mine ecosystem restoration and reconstruction on regional carbon sequestration and stock in the mine area is important for understanding the development of regional carbon sequestration potential under source-sink conversion. It can provide a theoretical basis for the local government and related departments to make ecological decisions to achieve the “double carbon goals” and promote regional sustainable development.

Therefore, we take the mining and metallurgical city of Huangshi City as a case and select four districts and one city under its jurisdiction as the study area. First, the regional carbon sink was estimated using multivariate datasets and ecological models. Then, the spatial and temporal evolution characteristics of NPP in the study area from 1990–2018 were systematically analyzed. Second, scenarios of climate change and land-use change were set up to study the effects of climate factors, landscape pattern evolution, and ecological restoration of mining areas on regional carbon storage to elucidate the carbon sink effect of ecological restoration of mining areas. Meanwhile, this study evaluated the effect of multi-year vegetation reconstruction in the Huangshi City from the perspective of time-series NPP changes. The ecological restoration-related measures to enhance the carbon sink of the mining area in the study area in the context of “double carbon goals” were explored and sorted out. Moreover, new ideas are provided for ecological restoration, environmental management evaluation, and sustainable development of mining areas.

## 2. Material and Method

### 2.1. Study Area

**Figure 1:**
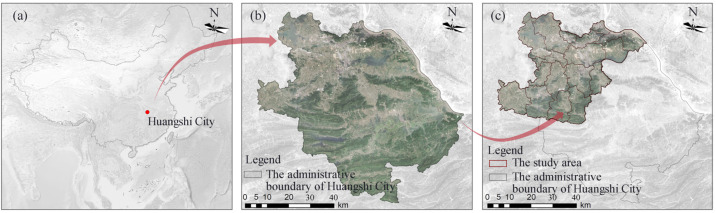
Location of Huangshi City (**a**), the administrative boundary of Huangshi City (**b**), and the study area (**c**).

Huangshi City is located in the southeast of Hubei Province, in the south bank of the middle reaches of the Yangtze River. The total land area is 4583 km^2^, with 4 districts, 1 county, and 1 city under its jurisdiction. As a traditional industrial base in China, Huangshi City has experienced long-term large-scale mining process, which has damaged environments. In recent years, Huangshi City has actively carried out ecological management of mines, such as Huangshi National Mine Park, which has planted more than 1.2 million acacias on the quarry, covering an area of 3.66 million m^2^, with a reclamation rate of 91%, making it the largest hard-rock reclamation base in Asia. However, the task of mine management is still arduous, and the existing mines and abandoned mines in the city currently occupy an area of more than 20,000 hm^2^, with 2075 surface and vegetation damages, 40 million m^3^ of mudflow formation, and more than 60,000 hm^2^ of pollution impact [18]. In this paper, five districts of Huangshi city with mining activities were selected as the study area, specifically including Huangshi port district, Xisaishan district, Xiaolu district, Tieshan district, and Daye city (Figure 1).

### 2.2. Data Source

We selected seven periods of 1990, 1995, 2000, 2005, 2010, 2015, and 2018 at 30 m spatial resolution land-use data (sourced from the website of Geographical Sciences and Resources, Chinese Academy of Sciences, http://www.resdc.cn, accessed on 13 April 2021). To ensure high quality and consistency, we combined historical images and field survey data information. The study focuses on the restoration of open-pit mining areas, so the mining land was extracted based on the original data classification, and finally, six types of land were obtained: arable land, forest land, grassland, urban construction land, mining land, and water (Table 1).

In addition, we acquired for summer and autumn cloud-free Landsat images for seven periods from 1990–2018, then performed pre-processing, such as radiometric calibration and atmospheric correction, to reduce atmospheric effects and possible errors in subsequent data calculations. NDVI data from 1990–2018 were calculated to provide basic input data for the subsequent NPP assessment.

The ground-based observation data of five meteorological stations (Wuhan, Jiayu, Yingshan, Lushan, and Huangshi stations) from 1990–2018 (sourced from the website of Meteorological Science Center, http://data.cma.cn/) and three radiation data stations (Wuhan, Hefei, and Nanchang stations) were obtained. The meteorological data were spatially interpolated in ArcGIS 10.4 to obtain annual mean temperature, annual total rainfall, and annual solar radiation raster data from 1990–2018.

### 2.3. Data Analyzing

#### 2.3.1. CASA Model

In this study, a modified CASA model (Equation (1)) was used to calculate vegetation net primary productivity [19]. The modified CASA model is a light energy utilization model based on remote sensing data, climate data (including temperature, precipitation, and total solar radiation), and vegetation type and soil type jointly driven [20]. The model uses meteorological data (temperature, precipitation, net solar radiation) in combination with existing regional evapotranspiration models to achieve the estimation of water stress factors, without the need for complex soil parameter calculations.

(1) NPP = APAR×ε

where NPP is the net primary productivity (gC·m^−2^), APAR is the absorbed photosynthetic active radiation (MJ·m^−2^·year^−1^), and ε is the light use efficiency (gC·MJ^−1^).

APAR depends on the total solar radiation (SOL) and the fraction of photosynthetically active radiation absorbed by the vegetation canopy (FPAR) and is described by Equation (2):

(2) APAR(x, t)=SOL(x, t)× FPAR(x, t)×0.5

where *t* is time (i.e., month), and *x* is spatial location (i.e., pixel). The constant 0.5 represents the proportion of solar effective radiation available to vegetation in the total solar radiation [21]. In this study, FPAR was estimated by Equation (3), as follows:

(3) $$\mathrm{FPAR}\left(x,t\right)=\frac{\mathrm{FPAR}{\left(x,t\right)}_{\mathrm{NDVI}}+\mathrm{FPAR}{\left(x,t\right)}_{\mathrm{SR}}}{2}$$

where FPAR(*x*, *t*)_NDVI_ and FPAR(*x*, *t*)_SR_ are FPAR calculated by NDVI (see Equation (4)) and SR (see Equation (5)) in *x* pixel and *t* month, respectively [18].

(4) $$\mathrm{FPAR}{\left(x,t\right)}_{\mathrm{NDVI}}=\frac{\left(\mathrm{NDVI}\left(x,t\right)-{\mathrm{NDVI}}_{i,\min}\right)\left({\mathrm{FPAR}}_{\max}-{\mathrm{FPAR}}_{\min}\right)}{\left({\mathrm{NDVI}}_{i,\max}-{\mathrm{NDVI}}_{i,\min}\right)}+{\mathrm{FPAR}}_{\min}$$

(5) $$\mathrm{FPAR}{\left(x,t\right)}_{\mathrm{SR}}=\frac{\left(\mathrm{SR}\left(x,t\right)-{\mathrm{SR}}_{i,\min}\right)\left({\mathrm{FPAR}}_{\max}-{\mathrm{FPAR}}_{\min}\right)}{{\mathrm{SR}}_{i,\max}-{\mathrm{SR}}_{i,\min}}+{\mathrm{FPAR}}_{\min}$$

The maximum and minimum values of FPAR were 0.950 (FPAR_max_) and 0.001 (FPAR_min_), respectively. NDVI*_i_*_,min_ and NDVI*_i_*_,max_ refer to the minimum and maximum values of NDVI for the land-use type *i* in month *t* (Table 2). SR*_i_*_,min_ and SR*_i_*_,max_ refer to the minimum and maximum values of SR for land-use type *i* in month *t* (Table 2). SR(*x*, *t*) is the simple ratio of NDVI in *x* pixel and *t* month and is calculated by NDVI(*x*, *t*) following Equation (6) [21]:

(6) $$\mathrm{SR}\left(x,t\right)=\frac{1+\mathrm{NDVI}\left(x,t\right)}{1-\mathrm{NDVI}\left(x,t\right)}$$

where NDVI(*x*, *t*) is NDVI in *x* pixel and *t* month.

The light use efficiency (ε) is the efficiency of vegetation in converting absorbed photosynthetic effective radiation into organic carbon [20]. It is mainly affected by temperature and moisture, as in Equation (7):

(7) $$\varepsilon \left(x,t\right)=T_{\varepsilon 1}\left(x,t\right)\times T_{\varepsilon 2}\left(x,t\right)\times W_{\varepsilon }\left(x,t\right)\times \varepsilon_{\max}$$

where $T_{\varepsilon 1}\left(x,t\right)$ and $T_{\varepsilon 2}\left(x,t\right)$ are temperature stress coefficients, $W_{\varepsilon }\left(x,t\right)\;$ is the moisture stress coefficient, and ε_max_ is the maximum light use efficiency as determined by the empirical method (Table 2). This ensures the reliability and availability of the data source on the one hand and simplifies the relevant parameters to a certain extent on the other hand, which enhances its practical operability. The model is operational, and the model accuracy has been validated [21].

#### 2.3.2. Statistical Analysis Methods

In this study, the data were all unified to 30 m spatial resolution, and we analyzed the spatial and temporal evolutionary characteristics of NPP data from 1990–2018 with the help of statistical tools, such as mean analysis, simple difference, and trend analysis.

#### 2.3.3. Scenario Setting

To investigate the effects of climate change and landscape pattern evolution on the regional carbon sequestration capacity, four scenarios were set up in this study, and the potential NPP of the study area in 2018 was calculated under different scenarios (Table 3). Among them, scenario 1 only excluded the impact of climate change on the NPP of the study area and calculated the impact of climate change and landscape pattern evolution on the regional carbon storage by combining the real NPP of the study area in 2000 and 2018 through the difference method [22,23]. The remaining three scenarios excluded both climate change and some land use changes and were compared with Scenario 1 separately to obtain the impacts of cropland change, forest land change, and non-restoration after mine development on regional carbon stocks.

## 3. Results

### 3.1. Validation of CASA Simulation Results

Regional-scale NPP data are difficult to obtain. Comparing the assessment results of this study with other similar studies, we were able to verify the accuracy and reasonableness of ecological model estimation [24]. Compared with other similar studies, the NPP of cropland, grassland, and forest land assessed in this study is within a reasonable range (Table 4) and can be used in subsequent studies [25,26], indicating that the regional NPP assessed based on the modified CASA model is more feasible in this study.

### 3.2. Analysis of NPP Spatial and Temporal Variation Characteristics

#### 3.2.1. Spatial Variation Characteristics of NPP

**Figure 2:**
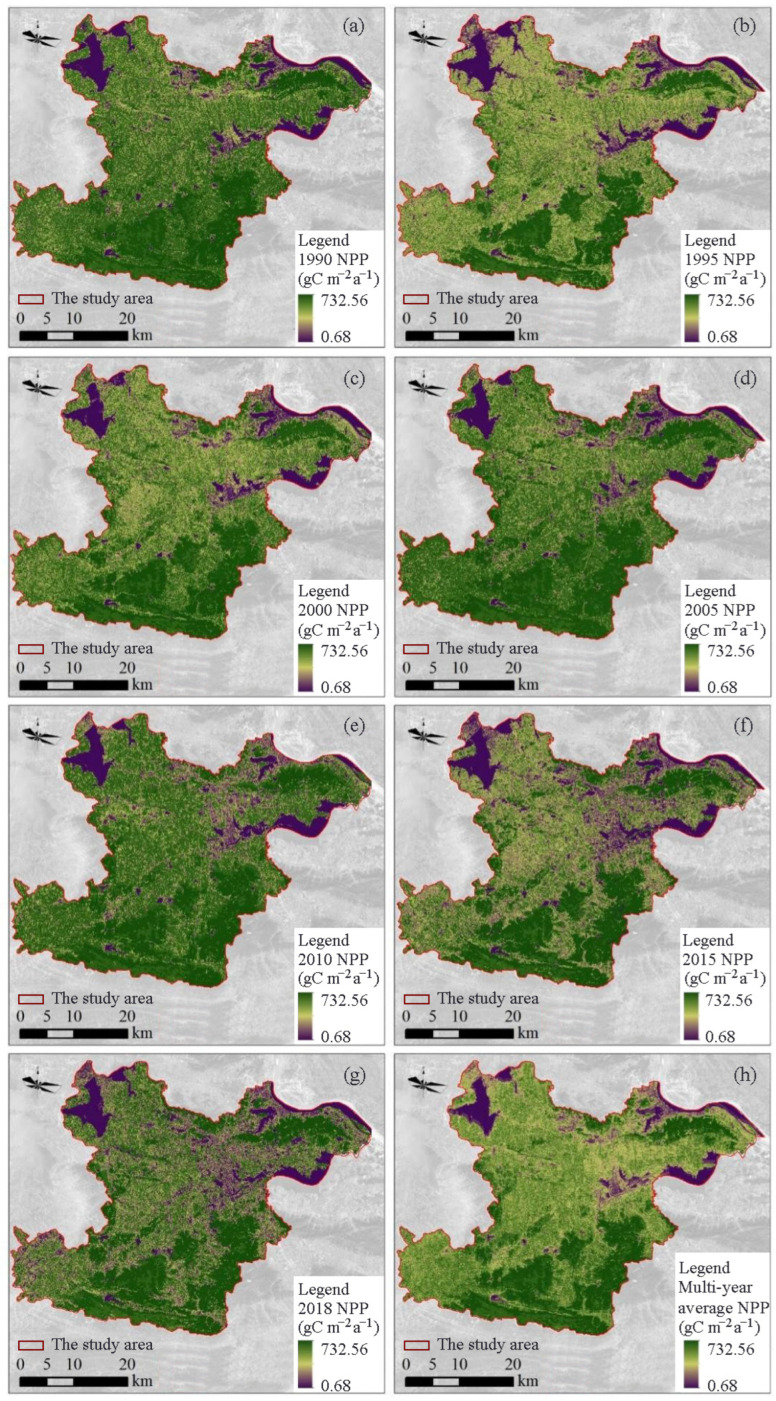
Spatial maps of NPP of the study area in 1990 (**a**), 1995 (**b**), 2000 (**c**), 2005 (**d**), 2010 (**e**), 2015 (**f**), and 2018 (**g**), and the average NPP (**h**) of the study area between 1990 and 2018.

The spatial distribution maps of NPP for the seven periods were evaluated by the modified CASA model (Figure 2), and the spatial distribution of NPP varied in the study area. The higher NPP areas were mainly distributed in the northeast and the southern mountainous areas, concentrated in the north of Huangshi Economic Development Zone in Huangjing Mountain, Jinhu Street Office, the southern part of Dajipu Town, and in the south of the study area in Lingxiang Town, Liurenba Town, and Yinzu Town.

The spatial distribution map of the multi-year average NPP was calculated based on the NPPs of the seven periods (Figure 2h). The lower NPP areas were in the central and northern parts of the study area and concentrated in Huangshi as well as the Daye city district. In addition, NPP is also lower in some scattered mining areas. These mining areas mainly include the Diqiao-Tieshan mining area in the north of the study area, Jinshandian town, Lingxiang town, Chengui town, Jinshandian mining area in Daye city, Tonglvshan mining area, Xiasifang mining area, Lingxiang iron ore mining area, and Tongshankou copper mining area in Daye city. The higher NPP areas were more concentrated, mainly in Dongfang Mountain, Huangjing Mountain, and the southern foothills of the study area. The distribution of NPP indicates that vegetation restoration in the southern of the study area is significant, the vegetation growth condition is better than that in the central and northern areas, and the carbon sequestration ability is strong.

**Figure 3:**
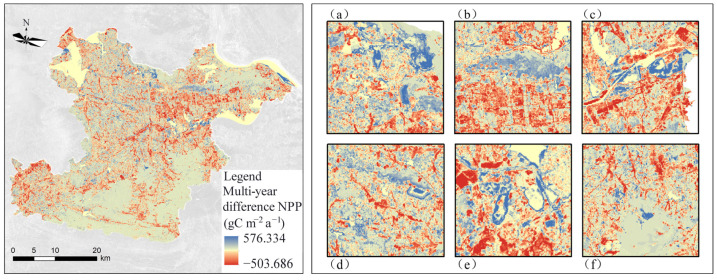
Distribution of NPP difference in Huangshi City from 1990 to 2018: (**a**) Tieshan-Huidiqiao mining area, (**b**) Huangjingshan mining area, (**c**) Sanli Qihu wetland, (**d**) Jinshandianzhen mining area, (**e**) Tonglushan mining area, and (**f**) Dajipu mining area.

The NPP difference in the study area between 1990 and 2018 was obtained by the simple difference method (Figure 3), with blue color indicating an increase NPP and red color indicating a decrease NPP. Compared with 1990, the NPP increased in most areas of the study area in 2018, but there were also some areas where the NPP decreased. Most of the areas with increased NPP were concentrated in the Tie Shan, Dong Fang Shan, and Huang Jing Shan areas in the north as well as along the foothills in the west and south. These areas are mainly hilly and mountainous, with more vegetation cover and less human interference, so NPP changed insignificantly in these areas. The areas with decreased NPP are mainly concentrated in the central urban economic development zone, and other areas with decreased NPP are scattered and concentrated in various towns. The main reason for the decrease of NPP in these areas is that most of the arable land, forest land, and grassland converted into urban construction land due to urbanization process, which destructed surface vegetation and decreased the vegetation coverage.

In addition, we extracted mining areas with a significant increase in NPP, mainly including Tieshan–Returning Bridge mining area, Huangjing Mountain, Tonglvshan mining area in Jinhu Street Office, Sanliqi Lake coastal waters wetland, Jinshandian iron ore mining area, Dajipu Longjiao Mountain–Xiasifang mining area, and Lingxiang iron ore mining area. The ecological restoration work in the mining area has achieved initial results. The re-greening project in the mining area has been carried out in an orderly manner, and the vegetation cover has increased, thus increasing NPP.

#### 3.2.2. Characteristics of NPP Temporal Variation

**Figure 4:**
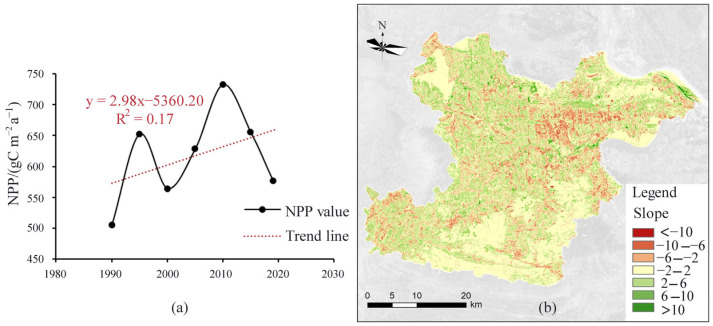
The NPP change trend of the study area from 1990 to 2018 (**a**), the spatial distribution of the NPP change trend from 1990 to 2018 at the pixel scale (**b**). Note: In order to quantify the dynamic of NPP, we used a least-square linear regression model to fit it. The change trend is described by the modeled slope, which is a of the y=ax+b. The red dotted line is the trend line of NPP, and the change trend is +2.98 gC m^−2^ a^−1^ per year.

The CASA assessment showed that the multi-year mean value of NPP in the study area was 616.60 (unit: gC m^−2^ a^−1^), and the mean values of NPP from 1990–2018 were 504.70, 652.71, 564.19, 628.53, 733.28, 655.82, and 576.95 (unit: gC m^−2^ a^−1^), respectively. The NPP change curve was obtained by trend analysis (Figure 4a), and the NPP increased slowly at a rate of 2.98 gC m^−2^ a^−1^ per year for 28 years. Overall, the increase in the average annual NPP of the study area indicates the enhancement of the regional carbon sequestration capacity. The carbon sequestration of the study area from 1990–2018 has obvious dynamics and has different dynamic characteristics in each period.

The spatial distribution of NPP dynamic from 1990 to 2018 was obtained by the trend analysis (Figure 4b). The areas with a significant increase in NPP were mainly the green part on the way, concentrated in the Tieshan–Returned Bridge mining area, the area around Huangjing Mountain, the Tonglvshan mining area, the wetlands in the waters of Sanliqi Lake, the mining area in Jinshandian Town, and the mining area in Lingxiang Town. It shows that the vegetation in these areas has recovered significantly, and the environment has improved significantly, and the vegetation is growing well. Therefore, the environmental management project of the mining area in the study area has achieved certain results, and the effect of the mine re-greening project is significant. The carbon sequestration of vegetation has increased to a certain extent compared with 1990, and the ecological benefits are significant. The red area in the figure shows that the national forest coverage declined significantly during 1990–2018, mainly concentrated around the economic development zone at the border of Daye and Huangshi city districts. Due to the influence of the industrialization process, a large amount of forest land and arable land here has been encroached on as construction land. The rest of the areas with reduced NPP are mainly scattered in various townships, where the number of people and the area of each township has increased due to the accelerated urbanization process, which has contributed to the reduction of carbon storage.

### 3.3. Analysis of Factors Influencing NPP Variation

#### 3.3.1. Analysis of the Influence of Climatic Factors on NPP Changes

**Figure 5:**
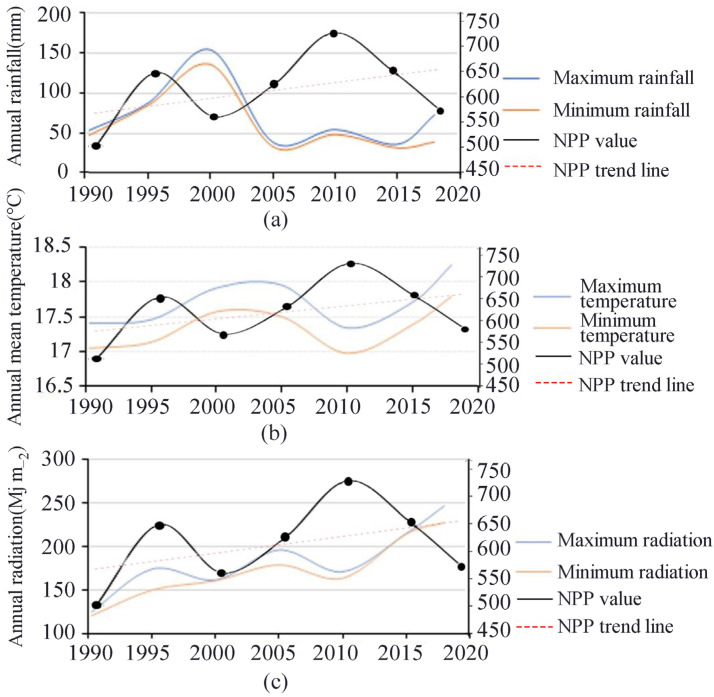
Changes in annual radiation (**a**) annual average temperature area (**b**) annual rainfall (**c**) of the study area.

Climatic factors have an important influence on NPP. Carbon sequestration and biomass accumulation are importantly linked to soil, water, and nutrient cycling as well as climatic conditions, while changes in light, water, and thermal conditions can have a significant impact on NPP in a region. In this study, the temporal trends of solar radiation, temperature, and precipitation in the study area from 1990–2018 were statistically analyzed (Figure 5), and the comparison revealed that the trends of radiation and temperature were closest to those of NPP. Although the spatial variation of solar radiation in the study area was small, it showed an increasing trend year by year from 1990–2018, and the comparison of the interannual trends of NPP and radiation revealed a certain correlation between them (Figure 5a), indicating that solar radiation can have some influence on regional NPP. In addition, the temperature variation in the study area was weak during 1990–2018 (Figure 5b) and remained stable, indicating that the temperature variation has a low degree of influence on NPP. Rainfall, a major influence in climate, plays a key role in NPP [27], which can increase the photosynthesis of plants. The spatial variation of rainfall in the study area is about 20 mm, varying east to west, with more in the east and less in the west. Under the influence of monsoon climate, precipitation generally decreases from east to west, and the spatial variation is not significant; however, the temporal variation is obvious, with more rainfall around 2000, and gradually decreasing and stabilizing thereafter. The multi-year average rainfall variation rate is larger in the north and remains basically stable in the south. Comparing the interannual variation trend of NPP (Figure 5c), we found that the rainfall was higher in 2000, but the NPP decreased. Excessive rainfall led to rainwater pooling and saturation of soil water content. Waterlogging conditions even occurred, resulting in excessive soil water content, which tends to cause plant root rot and is not conducive to plant growth, so NPP showed a decreasing trend. From 2005 to 2018, the average annual rainfall decreased, and NPP showed an increasing trend.

#### 3.3.2. NPP Dynamic and Land-Use Change

Combined with the land-use changes from 1990–2018 (Table 5), the land-use change can directly or indirectly affect NPP. During 1990–1995, the arable land decreased obviously with 14.47 km^2^; forest land and mining land increased, but forest land increased less. During 1995–2000, arable land and mining land increased slightly. During 2000–2005, arable land decreased substantially and mining land increased slightly. From 2005 to 2015, arable land continued to decrease greatly, forest land also showed an area reduction, and mining land increased substantially. During 2015–2018, arable land and forest land continued to decrease, while mining land gradually increased. From 1990 to 2018, arable land and forest land decreased by 106.17 km^2^ and 6.19 km^2^, respectively, and mining land increased. The NPP increased by 72.25 gC m^−2^ a^−1^ during this period, indicating that the ecological restoration work after the development of the mine area could compensate for the negative environmental effects caused by deforestation and field destruction to some extent.

### 3.4. Scenario Analysis of Carbon Sequestration Capacity in the Study Area

#### 3.4.1. Influence of Climate and Landscape Pattern Changes on Regional Carbon Storage

**Figure 6:**
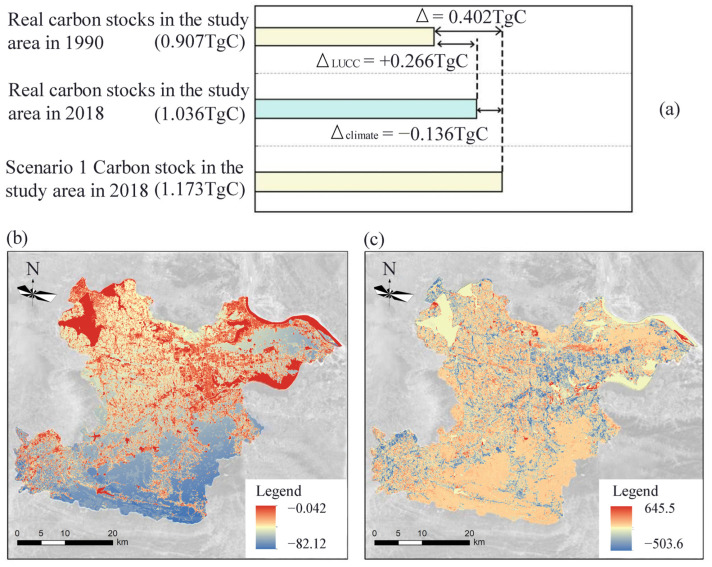
Scenario analysis for climate and landscape pattern changes (**a**), the impact of climate change on NPP (**b**), and the impact of landscape pattern evolution on NPP (**c**).

According to scenario 1, the potential NPP0 in 2018 was 652.698 gC m^−2^ a^−1^ and the annual carbon stock in the study area was 1.172 TgC (Figure 6a). The actual NPP in 2018 was 576.95 gC m^−2^ a^−1^ and the annual carbon stock in the study area was 1.036 TgC. Climate change caused a decrease of 0.136 TgC in the annual carbon stock in the study area. The actual NPP in 1990 was 504.702 gC m^−2^ a^−1^, and the annual carbon stock in the study area was 0.906 TgC. The evolution of the landscape pattern increased the annual carbon stock of the study area by 0.266 TgC sequestered carbon. It may be that under the influence of human activities, such as ecological restoration, the mining land is converted to green land with higher carbon storage. The carbon sequestration is greater than the negative effect caused by the increase of urban construction land and mining land, increasing regional carbon sequestration.

The spatial distribution map of NPP0 and the spatial distribution map of NPP2018 were analyzed for differences, and a map of the impact of climate change on regional carbon sequestration capacity was obtained (Figure 6b). The significant impact of climate change on NPP was concentrated in the foothills of the southern part of the study area. The loss of carbon sequestration caused by climate change was mainly due to the high altitude and complex terrain in the foothills. In addition, excessive rainfall and poor drainage in the foothill area caused serious waterlogging, which affected the normal growth of vegetation and led to the reduction of vegetation carbon sequestration capacity. Meanwhile, NPP dynamic was affected by some climatic factors, such as atmospheric CO_2_ concentration and atmospheric changes [28].

The spatial distribution map of NPP0 and the spatial distribution map of NPP1990 were analyzed by the differences analysis, and the map of the influence of landscape patterns on regional carbon sequestration was obtained (Figure 6c). The areas with the significant positive influence of landscape pattern on NPP are mainly concentrated in the Tieshan–Returning Bridge mining area, the southern foot of Huangjing Mountain, Daye Lake, and Sanliqi Lake ecological wetland, Tonglvshan mining area in Jinhu Street Office, and Chengui Town mining area, etc. The ecological restoration of mining areas can effectively improve NPP and promote regional carbon storage. The areas with significant positive effects of landscape pattern on NPP were mainly concentrated in the urban construction area in the central part of the study area, mostly due to the encroachment of arable land and forest land and the decrease of vegetation productivity caused by urban expansion.

#### 3.4.2. Impact of Vegetation Landscape Changes on Mine-Site Restoration and Regional Carbon Stock

**Figure 7:**
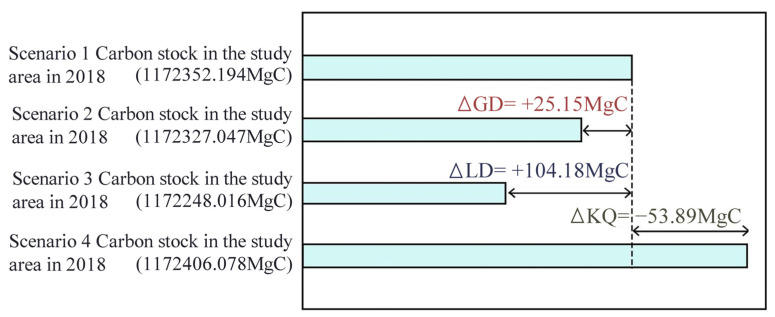
Effects of cropland change (GD), forest land change (LD), and ecological restoration of mining areas (KQ).

NPPs of the four scenarios were calculated by the modified CASA model, and the addition of cropland and forest land from 1990 to 2018 contributed to an increase in the regional annual carbon stock of 25.15 MgC and 104.18 MgC, respectively, while mine development could lead to a decrease in the regional annual stock of 53.89 MgC (Figure 7). Therefore, the new forest and cropland have a greater impact on the carbon sequestration, while the mine area will continue to release carbon without ecological restoration. Due to the implementation of the policy of returning farmland to forest, arable land protection, and mine restoration, the trend of decreasing arable land area has slowed down, the yield of arable land has increased, and some historical mining areas have been transformed into a forest, grassland, and arable land with high carbon sequestration capacity through ecological restoration. Although the arable land and forest land are reduced, the ecological environment of the mining area is gradually stabilized with the restoration process, leading to the overall improvement of the surrounding environment and promoting the improvement of the regional environmental carbon sequestration capacity. The development of mining areas will reduce the regional carbon sequestration, while urbanization will also gradually weaken the regional carbon sequestration.

Urban development transformed into a high-speed development stage with the increase of population growth and economic activities in the Huangshi City, leading to the intensification of human activities. The urbanization level is increasing, and regional transportation road network construction will all increase the proportion of construction land. The proportion of traditional industries is still gradually increasing in the transformation process, so it leads to a large amount of new mining land in the area of open pit mines on top of the original historical legacy, which leads to changes in the structure of the ecosystem and a decrease in the area and quantity of arable land and vegetation, which in turn leads to a decrease in the amount of carbon sequestration.

## 4. Discussion

### 4.1. Validation of the NPP Estimation of CASA

According to the CASA model estimates, annual NPP of the study area increasing slowly (Figure 4a). Compared with other similar studies [24], the NPP of cropland, grassland, and forest land assessed by CASA in this study is within a reasonable range (Table 3) and can be used in subsequent studies [25,26].

Despite the lack of experimental validation, we used correlation analysis to verify the accuracy of the estimates. The gap was not significant between our assessment structure and other models considered soil and respiration. The spatial and temporal dynamics of NPP estimated in our study for the Huangshi region are consistent with the related study by Liu et al. [29]. For example, Liu et al. used the CASA model to estimate the spatial and temporal variability of carbon dynamics in the study area, and they reported an increasing trend of NPP from 522.63 gC m^−2^ a^−1^ to 615.82 gC m^−2^ a^−1^ during 2000–2015 in the study area.

### 4.2. Spatial and Temporal Evolutionary Characteristics of NPP

The spatial distribution of NPP in the study area varied significantly from 1990 to 2018. The areas with medium-low carbon sequestration were mainly distributed in the central, northwest, southwest, and other topographically flat areas (Figure 2). The areas with high carbon sequestration were mainly distributed in the northeastern as well as the mountainous areas in the south. The NPP in the central and northern parts of the study area is extremely low and are concentrated in Huangshi as well as Daye city and some scattered mining areas. Macroscopically, the NPP of the study area showed a mild increasing trend; microscopically, while the NPP of the mining area increased significantly, indicating that the ecological restoration project increased the vegetation cover, stabilized and improved the ecological environment in the mining area, thus promoting the overall carbon sequestration capacity of the region (Figure 3).

NPP of the study area differenced obviously among periods (Figure 4). The NPP fluctuated between 1990 and 2018, with an overall insignificantly increasing trend. The areas with significant increases in NPP were concentrated in each mining area, indicating that the effective implementation of the mine ecological restoration project in the study area vigorously improved the ecological environment of the mining area, stabilized the mining ecosystem, and promoted the significant increase of NPP in the mining area (Figure 5).

### 4.3. Responses of NPP to Landscape Dynamics and Climate Changes

The evolution of landscape patterns influenced the regional carbon sequestration to a greater extent [30]. Land-use change increased carbon sequestration by 0.266 TgC, while climate change lost 0.136 TgC sequestration. Climate factors had a significant negative effect on NPP change [14,31], while landscape pattern evolution had a significant positive effect on NPP change. Due to the conversion of mining land into greenfield land with higher carbon sequestration as well as the increase in the area of arable land and forest land, carbon sequestration increased in the area of countervailing urban building land. Therefore, land-use changes promoted carbon sequestration (Figure 6), indicating that the environmental effect brought by ecological restoration is significant. The regional environment is stable, and the regional carbon sequestration capacity is steadily improved.

Changes in vegetation landscape and ecological restoration of mining areas have a significant impact on NPP [15]. Reforestation makes the largest contribution to carbon sequestration (Figure 7), while non-restoration of mining areas after mining promotes regional carbon release [32]. From 1990 to 2018, the contribution of new forest land to the regional annual carbon stock is 104.17 MgC, and the contribution of new arable land to the regional annual carbon stock is 25.14 MgC. This may be caused by the implementation of the policy of returning farmland to forest, protecting arable land, and mine rehabilitation. As a result, the decreasing arable land has slowed down, the yield of arable land has increased, and the ecological environment of the mine area has gradually stabilized with the restoration process. Thus, it led to the overall improvement of the surrounding environment, promoted the enhancement of regional environmental carbon sequestration, and facilitated the increase of carbon sink. The transformation of Huangshi’s urban development into a high-speed development stage, the proportion of construction land increases, new mining land is added, and the failure to restore the mining area in time after mining will lead to the reduction of regional carbon sequestration [33].

### 4.4. Ecological Restoration for Mining Areas

In summary, the following suggestions are made for the ecological restoration of mining areas and carbon sequestration regulation in Huangshi: (1) Ecological restoration of mining areas is the main increment of regional NPP, and ecological restoration of stocked mining areas should be continuously promoted regional carbon storage [10]. (2) Precise policies should be applied to the key areas of NPP reduction. Strictly control the urban growth boundary, especially the economic development area in the junction zone of Daye and Huangshi districts with the greatest degree of NPP reduction, strictly prohibit the destruction and occupation of the area and the surrounding mountains and arable land, and actively restore abandoned mines in the area. (3) Encouraging the restoration of mining areas to forest land and other ecological lands could contribute to carbon sequestration, while combining the governance of mining areas with the maintenance of the continuity and health of the regional ecological pattern and process could enhance the regional carbon storage [29]. (4) Establishing a dynamic monitoring mechanism for NPP in mining areas could provide timely feedback on the effectiveness of ecological restoration in mining areas. Optimizing the workflow of ecological environment management in mining areas in the future provides strategies for sustainable development of an ecological environment in mining areas and enhancement of carbon sinks [28].

## 5. Conclusions

This study systematically assessed and analyzed the net primary productivity (NPP) of vegetation in four districts and one city of Huangshi from 1990–2018. NPP was also used as an indicator to identify the carbon sequestration. The study focused on the impact of ecological restoration in mining areas to further provide a theoretical basis for ecological restoration and reconstruction in mining areas. Our results documented that (1) NPP in the study area from 1990–2018 shows an increasing trend and exhibits significant spatial heterogeneity; (2) the significant increase of NPP was mainly in the restored mining areas, indicating that the ecological restoration of mining areas can effectively improve the regional carbon sequestration capacity; (3) from 1990 to 2018, climate change contributed to a decrease of 0.136 TgC, while landscape pattern change contributed to an increase of 0.266 TgC; and (4) the restoration and reconstruction of mine ecosystems is an important way to achieve carbon neutrality in Huangshi in the future, and the changes of NPP under different ecological restoration modes are significantly different.

## Figures and Tables

**Table 1:** Land use Classification.

Land Use Types	Definition
Cropland	Land for growing crops, including ripe cultivated land, newly opened land, recreational land, rotational land, grass field rotation cropland; land for growing crops mainly agricultural fruits, agricultural mulberry, agricultural forestry; beach land and sea shoals that have been cultivated for more than three years.
Woodland	Forestry land with trees, shrubs, bamboos, coastal mangroves, and other forests.
Grassland	Grasslands with herbaceous plants covering more than 5% of the land, including scrub grasslands mainly for grazing and sparse forest grasslands with a depression of less than 10%.
Waters	Land for natural land water and water conservancy facilities.
Urban construction land	Large, medium, and small cities and counties and towns above the built-up areas and rural settlements independent of the towns.
Mining land	Factories, mines, industrial zones, oil and salt fields, quarries, unused land, and special land.

**Table 2:** Parameters of the CASA model for different land use types.

Land Use Type	NDVI_max_	NDVI_min_	SR_max_	SR_min_	ε_max_
Cropland	0.7994	0.0765	14.393	1.166	0.729
Woodland	0.8979	0.0765	18.793	1.166	0.985
Grassland	0.6653	0.0765	12.576	1.166	0.429
Water	0.5044	0.0765	8.97	1.166	0.429
Urban construction land	0.5044	0.0765	8.97	1.166	0.429
Mining land	0.5044	0.0765	8.97	1.166	0.429

Note: NDVI_min_ and NDVI_max_ refer to the minimum and maximum values of NDVI for each land use type. SR_min_ and SR_max_ refer to the minimum and maximum values of SR for each land use type. The ε_max_ refers to the maximum value of light use efficiency.

**Table 3:** Scenario setting and analysis.

Scenario	Meaning
Scenario 1: The climate factors (precipitation, temperature, and solar radiation) are fixed in 1990, and the study area 2018 NPP (NPP0) of the constant climate scenario is assessed with the help of CASA using the real 2018 NDVI and land-use data. By analyzing the relationship between NPP0, true NPP2000 in 2000, and true NPP2018 in 2018 and multiplying by the area of the study area, we were able to obtain the impact of climate change and landscape pattern evolution on the carbon stock in the study area from 1990–2018.	Impacts of climate change on carbon stocks in the study area: Δclimate = (NPP2018 − NPP0) × area Impact of landscape pattern evolution on carbon stocks in the study area: Δlucc = (NPP0 − NPP1990) × area
Scenario 2: The climate factors (precipitation, temperature, and solar radiation) are fixed in 1990 while assuming no change in the new cropland and its corresponding NDVI, and the 2018 NDVI and land-use data with constant cropland are obtained. The 2018 NPP (NPPGD) of the study area for the constant climate, constant cropland scenario was assessed with the help of CASA. By analyzing scenarios one and two, the impact of cropland changes on carbon stocks in the study area from 1990–2018 can be obtained.	Impact of arable land change on carbon stock in the study area: ΔGD = (NPP0 − NPPGD) × area
Scenario 3: The climate factors (precipitation, temperature, and solar radiation) are fixed in 1990 while assuming no change in the additional forest land and its corresponding NDVI, and the forest land constant 2018 NDVI and land-use data are obtained. The 2018 NPP (NPPLD) of the study area for the constant climate and constant forest land scenario was assessed with the help of CASA. By analyzing scenarios one and three, the impact of woodland change on carbon stock in the study area from 1990–2018 can be obtained.	Impact of forest land changes on carbon stock in the study area: ΔLD = (NPP0 − NPPLD) × area
Scenario 4: The climate factors (precipitation, temperature, and solar radiation) are fixed in 1990 while assuming no change in the developed mine area and its corresponding NDVI, and the 2018 NDVI and land-use data for the mine area are obtained for the constant mining area. The 2018 NPP (NPPKQ) of the study area for the constant climate, constant mine scenario was assessed with the help of CASA. By analyzing scenarios one and four, it was possible to obtain the impact of mining on carbon stocks in the study area from 1990–2018.	Impact of mining on carbon stock in the study area: ΔKQ = (NPP0 − NPPKQ) × area

**Table 4:** Comparison of NPP estimated value and actual measured value.

Research Method	NPP Values and Measured Values for Different Vegetation Types/(gC m^−2^ month^−1^)			Tine	Study Area
	Cropland	Forest Land	Grassland		
This study CASA	397.55–548.07	459.27–644.26	430.95–624.59	1990–2018	Huangshi, Hubei Province
CASA	390	382–956	405	2003–2012	Shaanxi Province
MOD17	-	586.85–606.31	541.31	2000–2015	The Wuling Mountains
Measured value	239–760	250–2500	100–727	-	China

**Table 5:** Change of land use area and NPP from 1990 to 2018.

Tine	Major Land Use Area Changes (km^2^)			NPP Changes (gC m^−2^ a^−1^)
	Cropland	Forest Land	Mining Land	
1990–1995	−14.47	1.25	2.97	148.01
1995–2000	4.98	−2.65	0.75	−88.52
2000–2005	−16.95	−1.58	6.36	64.34
2005–2010	−51.92	−1.52	26.50	104.75
2010–2015	−29.81	−3.41	24.11	−77.46
2015–2018	2.0	1.72	7.85	−78.87
1990–2018	−106.17	−6.19	68.54	72.25

## Data Availability

Not applicable.
